# Twin Pregnancy in Brazil: A Profile Analysis Exploring Population Information from the National Birth E-Registry on Live Births

**DOI:** 10.1155/2018/9189648

**Published:** 2018-11-01

**Authors:** Danielly S. Santana, Renato T. Souza, Fernanda G. Surita, Juliana L. Argenton, Cleide M. Silva, Jose G. Cecatti

**Affiliations:** ^1^Department of Obstetrics and Gynecology, University of Campinas School of Medicine, 101 Alexander Fleming Street, 13083-891 Campinas, Brazil; ^2^Statistics Unit, University of Campinas School of Medicine, 101 Alexander Fleming Street, 13083-891 Campinas, Brazil

## Abstract

Birth records as SINASC (Brazilian Live Birth Information System) are highlighted in uncommon conditions such as twin pregnancy whose prevalence rarely exceeds 2 to 3% of the total number of births. The objective of this study was to assess the prevalence of twin pregnancies in Brazil and their maternal and perinatal characteristics using data from the national birth e-Registry. All births in Brazil from 2011 to 2014 were assessed. Prevalence of twin pregnancies per region was assessed and correlated with the Human Development Index (HDI). Sociodemographic and obstetric factors and main perinatal outcomes were assessed for the first and second twin, in comparison to singletons, and the second twin compared to the first twin, with PR and 95%CI. A multiple logistic regression analysis was conducted to identify factors independently associated with a low 5-minute Apgar score in twin pregnancies. Twin pregnancy occurred in 1.13% in Brazil, with a higher prevalence in regions with a higher HDI. It was associated with a complete higher level of education (22.9% versus 16.3% for singles) and maternal age > 35 years (17.5% versus 11.4% for singles). Preterm birth <32 weeks (prevalence ratio-PR 12.13 [11.93 – 12.33]), low birth weight (PR 17.8 [17.6-18.0] for the first and PR 20.1 [19.8-20.3] for the second twin), and low Apgar score (PR 2.9 [2.8-3.0] for the first and PR 2.7 [2.6-2.8] for the second twin) were the most important perinatal outcomes associated with twin pregnancies. A 5-minute Apgar score < 7 among twins was associated with inadequate prenatal care, extreme preterm birth, vaginal delivery, intrapartum cesarean, and combined delivery. Twin pregnancy in Brazil is associated with worse perinatal outcomes, especially for the second twin.

## 1. Introduction

In low-income countries, vital statistics systems are insufficient or nonexistent. In these cases, population-based samples are used. Health records lack continuity, making it impossible to monitor conditions and evaluate the effectiveness of interventions [1–4]. Vital statistics are a form of national surveillance for health events, contributing to the creation of diverse population-based indicators. The cause of death, associated factors, and knowledge on sociodemographic characteristics of the population allow an analysis of the health situation and guide public health prevention and intervention strategies [1–4]. E-Registries are electronic information systems for vital health data storage. Birth e-Registries are specifically aimed at unifying information on individuals from preconception to the postpartum period and including newborn and child health data. Such records are an emerging opportunity for maternal healthcare researchers. However, middle- and low-income countries have failed to provide the collection, analysis, and notification of health data, resulting in information that is often incomplete and fragmented [5, 6].

Birth records are of importance to study uncommon conditions that are hardly well-evaluated in the analysis of population-based samples. Twin pregnancy is one of these conditions, since its prevalence rarely exceeds 2 to 3% of the total number of births [7, 8]. Although wide population-based evaluations are lacking, twin pregnancy is theoretically known for its higher risk of maternal, fetal and perinatal complications [9–14]. In addition, in the current proposal it was important to study not only the prevalence of twin pregnancy and its perinatal outcomes, but also the association between these results with fetal presentation, onset of labor, and delivery route that are intimately linked to obstetric practice, both in Brazil and around the world, and apparently differ from evidence-based recommendations [15].

The aim of the current study was to evaluate maternal and perinatal characteristics of twin pregnancies in comparison to single pregnancies, from information available in the Brazilian Live Birth Information System (SINASC) database, determining its prevalence in diverse Units of the Federation (UF), per region and Human Development Index (HDI), and identifying sociodemographic and obstetric characteristics associated with twin pregnancy. Another purpose of the study was to evaluate perinatal outcomes of each twin compared to singleton newborns, comparing the second to the first twin, and assessing whether perinatal outcomes were modified by presentation of the first twin and whether the delivery route modified newborn vitality.

## 2. Materials and Methods

The current study corresponds to a planned cross-sectional secondary analysis of birth data contained in the SINASC database in Brazil. It is an electronic registration system of the Ministry of Health, developed by the SUS Computer Science Department (DATASUS), and aimed at gathering epidemiologic information on live newborn infants across the national territory. Data is collected in a cross-sectional manner at the time of birth. It was implemented in 1990 by the Ministry of Health in all Units of the Federation. Since that date, it carries out continuous registration, with monthly consolidation of liveborn infants based on completion of the Certificate of Live Birth (CLB), a standardized document arranged in a single numerical sequence that is distributed in three copies to the whole country. The first copy must be filled out by the notification units: health departments, when delivery occurred in a hospital setting or other health institute, or Civil Registration Office in case of home deliveries [16, 17]. This document is mandatory and fundamental for each individual to receive the birth certificate. After filling out the CLBs, they are gathered, reviewed, and processed, with the creation of a Birth Registry, available among vital statistics on the DATASUS website [16].

CLB contain the following information: birth data (date, place, health facility, district, and municipality), maternal data (age, marital status, school education, ethnicity, occupation, number of liveborn and stillborn children, and city of residence), pregnancy and delivery data (gestational age, type of pregnancy, single or multiple, parity, number of previous cesarean sections, type of delivery, onset of labor, number of prenatal visits, and place of delivery), and newborn data (sex, Apgar score at one and five minutes, weight, presentation, and congenital anomalies). There are two versions of the CLB. The most recent version was implemented after 2010. Changes in data composition were included, granting access to more information of a better quality [16]. That is the reason why consolidated data for the years 2011 to 2014 were chosen for the current analysis. Data was more recent and complete, with the use of a new version of the CLB.

Technical resources for the creation of a database system changed over time until a program was introduced. This program allows the performance of tabulation via the Internet, which actually represents the migration of records to an electronic platform. It also allows for data selection and organization, according to research purposes, and associates tabulations to other resources such as maps and graphs [16].

**Figure 1:**
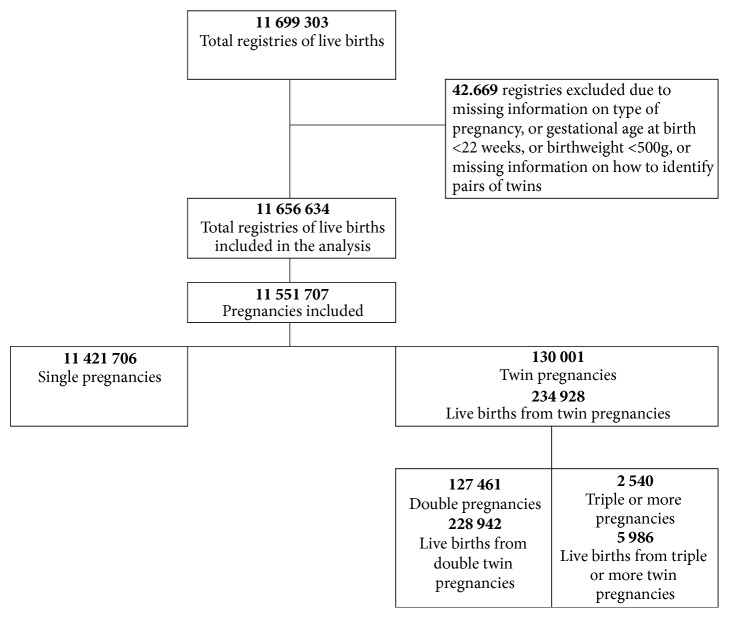
Flowchart for identification of twin pregnancies and live births for analysis, Brazil, SINASC 2011-2014.

For the current analysis, SINASC data from 2011 to 2014 were used. Data was compiled in a single database with information on a total of 11 699 303 live births. Excluded were certificates that had no information about the type of pregnancy, identification of pairs of twin siblings, gestational age less than 22 weeks, and birth weight below 500g, thus obtaining 11 656 634 live newborn infants. Of these, 234,928 were live births from twin pregnancies (228,942 twin births and 5,986 triplet births or other higher order births) and 11 421 706 were single live births (Figure 1). Liveborn infants originating from twin pregnancies were identified by record linkage procedures applied to the database, using information on maternal date of birth, newborn infant date, time of birth, and CLB number.

The prevalence of twin pregnancy in Brazil was identified per regions, states, and state's HDI. For characterization of each federal unit per HDI, the HDIM ranking of 2014 was used. It is an analysis based on the National Household Sample Survey that is published annually [18]. To analyze the prevalence of twin pregnancies per regions and states, Cramer's V Coefficient was used. Cramer V was indicated to evaluate the association between bidimensional conditions in populations. This coefficient indicates a weak or nonexistent association at a value between 0 and 0.1, a low association at a value between >0.1 and 0.3, a moderate association at a value between >0.3 and 0.5, and a strong association when >0.5 [19]. To evaluate the rates according to the HDI, a correlation analysis with Spearman's coefficient was conducted to test a linear association between HDI and the prevalence of twin pregnancy [20]. Then sociodemographic and obstetric characteristics of women were evaluated, in comparison to twin and single pregnancies, using Cramer's V Coefficient. For the three analyses, pregnancy (woman) was considered the unit of analysis.

Perinatal outcomes of twin pregnancies were evaluated. For the variables gestational age at birth, onset of labor, and type of delivery, the unit of analysis was the pregnancy (woman), comparing twin pregnancies to single pregnancies. For the variables birth weight, fetal presentation, presence of malformations, and Apgar score at 5 minutes of life, the newborn infant was considered the unit of analysis. Results were presented separately for the first twin, second twin, and newborn infants from single pregnancies. Comparisons were made in the following manner: between the first-born twin and singleton, between the second-born twin and singleton, and between the second-born and first-born twin. These results are shown as the Prevalence Ratio with their respective 95% confidence intervals. The first-born and second-born (and third, fourth, etc.) twins were identified using the sequential CLB number, after identifying pairs by a record linkage process. When fetal death of one twin occurred, it was not possible to identify which twin had died by the CLB. The twin who was born alive was therefore considered to be the first-born twin. The occurrence of some adverse perinatal outcomes according to the presentation of the first twin and low 5-minute Apgar scores depending on the route of delivery were evaluated only for twin pregnancies. For these evaluations, Cramer's V Coefficient was also used.

Finally, unconditional multiple logistic regression analysis was carried out. The aim was to identify factors independently associated with 5-minute Apgar scores < 7. For this analysis, Apgar scores were evaluated in each twin. Therefore, three sets of situations of negative results relative to Apgar scores were created: (1) both twins had 5-minute Apgar scores <7; (2) only the first twin had a 5-minute Apgar score < 7, and (3) only the second twin had a 5-minute Apgar score <7. Pregnancy was considered the reference where both newborn infants had 5-minute Apgar scores ≥ 7. All the remaining sociodemographic and obstetric variables were tested as predictors in the multiple analysis models.

For this analysis, all statistical procedures were performed with SAS software (version 9.4). Results were considered significant, interpreting Cramer's V Coefficient for values higher than 0.3. This association test was chosen because there was no need to carry out formal statistical tests. Due to the large size of the population, all p values would have been significant [20].

Concerning ethical aspects of the study, data were obtained from the Internet, as previously mentioned. The SINASC, the Brazilian electronic Birth Registry was used. It contains birth data without subject identification. This information and database are of public domain and therefore informed individual consent was not required. Nevertheless, ethical principles in human research contained in the Declaration of Helsinki were upheld. The study followed a detailed protocol and statistical analysis plan, performed by skilled statisticians, using adequate programs and techniques for this purpose. The identity of the subjects was kept confidential. No funding was available for this analysis that was performed under the tasks of the first author covered by a personal fellowship grant from the Brazilian Capes.

## 3. Results

**Figure 2:**
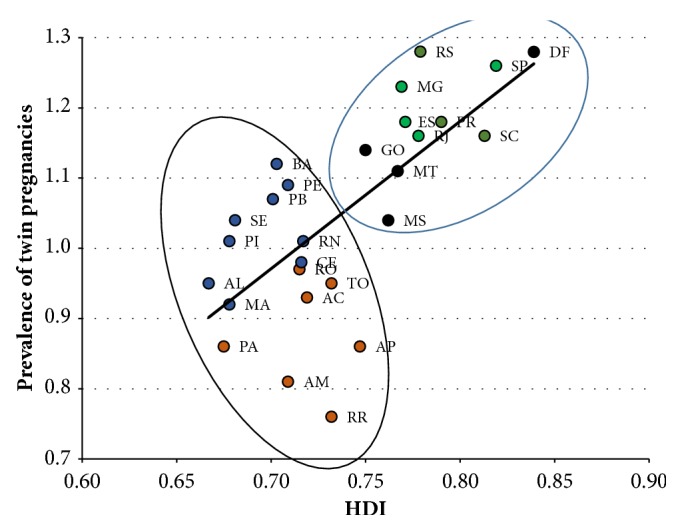
Spearman correlation between HDI-2014 and the prevalence of twin pregnancies in Brazilian states, SINASC 2011-2014. (The Spearman correlation coefficient for North and Northeast regions is -0.24, while for the southern, southeastern, and midwestern regions it is 0.71; the Spearman correlation coefficient for the whole country is 0.69.)** Legend for color circles: **red: states from the North region; blue: Northeast; black: Midwestern; green: Southeast; orange: South region.

During the period analyzed, twin pregnancies occurred in 1.13% of pregnancies in Brazil. The region with the highest prevalence of twin pregnancy was the Southeast (1.23%), followed by the Southern region (1.21%), Midwest (1.14%), Northeast (1.03%), and North (0.86%). The Federal District and Rio Grande do Sul (1.28%), São Paulo (1.26%), and Minas Gerais (1.23%) were the federation units that had the highest prevalence of twin pregnancies, as shown in Table 1. In Brazil, there seems to be a linear direct relationship between increased HDI and the prevalence of twin pregnancy, with a correction coefficient of 0.69. However, visualization of plotted points per state suggests the existence of two groups that behave in a distinct way in this correlation: one group, formed by only the Southern, Southeastern, and Midwestern regions, with higher HDI, shows a clear positive correlation between HDI and prevalence of twin pregnancies (Spearman's correlation coefficient of 0.71). In contrast, the other group, formed by states of the Northern and Northeastern regions, shows a clear negative correlation, and twin pregnancies decrease with increasing HDI (Spearman's correlation coefficient of -0.24) (Figure 2).

Regarding the sociodemographic and obstetric characteristics shown in Table 2, using Cramer's V Coefficient, none of the factors analyzed had a moderate or strong association, when twin and single pregnancies were compared. However, it was observed that twin pregnancy had proportionally more women in the group with a complete higher level of education (22.94% in twin pregnancies; 16.29% in single pregnancies) and age over 35 years (17.55% in twin pregnancies, 11.39% in single pregnancies). The differences between both groups were less evident for marital status, ethnicity/skin color, parity, prenatal visits, and place of birth.

Table 3 shows that twin pregnancies had a higher proportion of preterm births (53.57% versus 10.56% in single pregnancies), especially births at less than 32 weeks (PR 12.13, 95%CI 11.93–12.33). Labor induction was proportionally higher in single pregnancies (32.34%) than in twin pregnancies (11.02%), and cesarean section was the most common type of delivery among twin pregnancies (81%; PR 3.38, 95%CI 3.33–3.42). Twin pregnancy is directly related to low birth weight (58.28% for the first twin, 61.19% for the second twin and 7.28% for singleton newborn infants), representing a risk of LBW that is estimated to be 18 to 20 times higher among twins. A higher occurrence of noncephalic presentation was observed among twin pregnancies, especially for the second twin (risks estimated to be 8 to 10 times higher), occurrence of congenital malformations (risks estimated to be 40 to 50% higher), and 5-minute Apgar score <7 (risks estimated to be 2.7 to 2.9 times higher).

Considering only twin pregnancies, when the first twin is not in cephalic presentation, there seems to be a lower proportion of labor induction (6.52%) and a higher occurrence of cesarean section. Gestational age at birth and Apgar scores do not seem to be associated with fetal presentation (Table 4). There was a weak association between 5-minute Apgar score <7 and delivery route. For the first twin, there was a higher frequency of 5-minute Apgar score <7 when delivery was vaginal for both twins (6.57%) or vaginal for the first twin and cesarean section for the second twin (combined, 3.86%). However, this result is more evident in the second twin (7.54% when vaginal delivery was performed in both and 12.86% when vaginal delivery was performed in the first twin and cesarean in the second) (Table 5).

Table 6 shows that multiple analysis identified factors independently associated with 5-minute Apgar score <7 for both twins: number of prenatal visits < 7 (OR 2.37), preterm birth, especially extreme preterm birth (OR 30.77), and vaginal delivery for both twins (OR 3.44). Intrapartum cesarean delivery was associated with 5-minute Apgar scores <7 for both twins (OR 1.26) and for the second twin alone (OR 1.24). Cesarean delivery for the second twin was associated with 5-minute Apgar scores <7 for the second twin (OR 16.27).

## 4. Discussion

The few studies evaluating the occurrence and characteristics of twin pregnancies in Brazil show that rates range from 0.9 to 2.4%, quite close to the prevalence found in the current analysis [21–23]. One of them, using the SINASC database from 2003-2014, identified a twin birth prevalence of 1.19% [24]. In the current study, a higher prevalence was observed in places with a better HDI. A larger number of assisted reproductive technologies (ART) performed in these regions may possibly justify this finding. Assisted reproduction is a factor directly related to the increase in twin pregnancy in the last decades [7, 9]. According to the Assisted Reproduction Registry of Latin America, in 2013 Brazil had the largest number of registered institutes and ART procedures performed [25]. The country currently has 65 registered institutes for assisted reproduction: 38 in the Southeastern, 13 in the South, 8 in the Midwest, 6 in the Northeast, and none in the North. This seems to coincide with a higher proportion of twin pregnancies in regions with a higher HDI [26].

The higher prevalence of women with a complete higher level of education among twin pregnancies is not a condition directly related to its occurrence. It is associated with two major risk factors: a higher maternal age in this population, who may have delayed childbearing, resulting in physiological ovarian hyperstimulation; or ovarian failure in these women, who may require techniques for assisted reproduction [8, 27]. Another obstetric condition found was the lower number of prenatal visits (<7) independently associated with worse neonatal outcomes, with a risk 2.3 times higher for both fetuses of low 5-minute Apgar score.

Perinatal outcomes are impressive: preterm birth and low birth weight, well-known as associated with twin pregnancies, and perinatal mortality were highly prevalent among them [7, 10, 12]. Preterm birth occurred in 50% of twin births, a prevalence rate that was almost fivefold higher than in single pregnancies. When analyzing pregnancies of less than 32 weeks, the risk was 12 times higher than in single pregnancies [13, 28–30]. On multivariate analysis, preterm birth is associated with 5-minute Apgar score <7. When gestational age <32 weeks, the risk increases 30 times for both twins. At gestational age 32 to 36 weeks, the risk increases 2.5-fold. There are no available data from other studies to permit a comparison with these results, although diverse studies confirm the association between preterm birth and worse perinatal outcomes [11, 13, 31]. Low birth weight was 17 times more common in the first twin when compared to singleton newborn infants and 20 times more common in the second twin considering the same comparison group. These risks were higher than those previously reported [11, 13, 14, 32, 33]. Furthermore, in this analysis, we observed that the risk of congenital malformation was 1.5 higher among twins.

A high proportion of twin pregnancies experienced no onset of labor. This may be due to the fact that twin pregnancy is associated with diverse maternal and fetal complications, and then therapeutic cesarean delivery may thus be required. Another considerable percentage of women, even higher than observed in single pregnancies, experienced spontaneous labor possibly due to overdistension of uterine muscle fibers or premature rupture of membranes [28, 34, 35]. Although 40% of twin pregnancies had spontaneous labor, more than 80% underwent cesarean delivery. Other studies show that cesarean delivery is free from perinatal risks when the first twin is in noncephalic presentation, although cesarean delivery is still very common in twin pregnancies at all. Globally, the rates of cesarean delivery range from 34% to 82% in twin pregnancies [15, 21, 36].

Some studies have shown that a 5-minute Apgar score <7 is associated with twin pregnancies. In the current analysis, it was not different: a 2-fold higher risk was observed in twins in comparison to single pregnancies [13]. It was observed that vaginal delivery for both twins, intrapartum cesarean delivery and vaginal delivery for the first twin followed by cesarean delivery (combined), had lower Apgar scores for twins, mainly for the second twin. Intrapartum cesarean delivery and combined delivery (vaginal delivery followed by cesarean delivery for the second twin) is known to be associated with worse perinatal outcomes, this second condition associated with a prolonged delivery interval between the first and the second twin with worse outcome, with higher prevalence of metabolic acidosis for the second twin [37]. When the first twin is in cephalic presentation vaginal delivery is safe, although it is known that obstetric care is essential for perinatal outcome. A well-trained staff is key to perform procedures necessary especially for the second twin [37–39]. Analysis of this Brazilian population, however, showed that vaginal delivery occurred in 10.5% of pregnancies in which the first twin was not in cephalic presentation. Vaginal delivery followed by cesarean delivery for the second twin occurred in only 1.27% of pregnancies. Since the current analysis proposed to assess the quality of obstetric care relative to presentation of the first twin and delivery route, some of the results found may suggest inadequate care, according to the most recent evidence available. This includes, for instance, labor induction at a rate of 6.5% when the first twin was not in cephalic presentation and the extremely elevated general cesarean rate in multiple pregnancies. In addition, there was a proportion of deliveries that occurred outside of the hospital. These situations may indicate that obstetric care was far from that recommended [15, 40, 41]. The prevalence of congenital malformation is approximately 1.5 times higher in twin pregnancy compared with singletons, contributing to increase the risk of adverse outcomes.

The current study is important at a national level, exploring twin pregnancies at population level for the whole country. However, some limitations may be recognized. A careful and detailed data quality control was not really possible in a study like this, using big data from a national birth e-Registry. In addition, there are some variables whose information was not well collected, with a large number of missings, as is the case for the onset of labor, for instance. When using the data collection form created specifically to assess perinatal outcomes of liveborn infants, some conditions could not be evaluated. Fetal death was one condition, since the CLB is only completed for live births. There is also no information about the results of newborn progress. Conditions such as neonatal ICU admission or perinatal death were not properly evaluated. No data on chorionicity is included in the forms. Although it is well-known that chorionicity is associated with fetal complications, this association could not be identified. In addition, it would be interesting to have information on pregnancies resulting from assisted reproductive technologies such as IVF. However, unfortunately, the database comes from the certificate of live birth officially adopted in the whole country and does not contain this information. This could limit the power of associations identified.

The present results were generated from national population-based data and indicate the need for deeper analysis. Further multicenter studies involving large populations are required to obtain this data. The reasons for such a high rate of cesarean delivery and for the association between lower 5-minute Apgar scores and vaginal delivery for both twins should also be clarified. These issues deserve a specific approach, with designs aimed at elucidating factors associated with these results.

## 5. Conclusion

Many characteristics of twin pregnancies in Brazil are comparable to those observed worldwide. Twin pregnancy occurs in women of more advanced age or in locations that have better socioeconomic conditions due to its association with the use of assisted reproductive technologies. The condition is associated with a number of perinatal complications, with worse results for the second twin in particular. Some results, however, were surprising. Low birth weight was shown to be much more prevalent than in the literature. The 5-minute Apgar scores were consistently lower for twins and mainly for the second twin. Considering that the results obtained represent the whole population of newborn infants in Brazil, this information may help public health policy makers develop specific recommendations for healthcare protocols for twin pregnancies and for surveillance of more common complications and other conditions associated with them.

## Figures and Tables

**Table 1:** Prevalence of twin pregnancies in Brazil according to regions and states, Brazil, SINASC 2011-2014.

**Regions and states **	**Twin pregnancy**	**Single pregnancy**	**Cramer V**	HDI^**∗**^
	**n (%)**	**n (%)**		
**Midwestern region**	**10 581 (1.14)**	**914 414 (98.86)**	0.0075	
Federal District	2 572 (1.28)	197 913 (98.72)		0.839
Mato Grosso	2 322 (1.11)	207 046 (98.89)		0.762
Mato Grosso do Sul	1 752 (1.04)	166 501 (98.96)		0.766
Goiás	3 935 (1.13)	342 954 (98.87)		0.750
**Northeast region**	**34 077 (1.03)**	**3 258 671 (98.97)**	0.0071	
Alagoas	1 954 (0.95)	204 121 (99.05)		0.667
Bahia	9 088 (1.12)	803 939 (98.88)		0.703
Ceará	4 917 (0.98)	497 924 (99.02)		0.716
Maranhão	4 236 (0.92)	454 561 (99.08)		0.678
Paraíba	2 427 (1.07)	224 127 (98.93)		0.701
Pernambuco	6 146 (1.09)	556 947 (98.91)		0.709
Piauí	1 959 (1.01)	192 413 (98.99)		0.678
Rio Grande do Norte	1 900 (1.01)	186 719 (98.99)		0.717
Sergipe	1 450 (1.04)	137 940 (98.96)		0.681
**North region**	**10 726 (0.86)**	**1 230 415 (99.14)**	0.0058	
Acre	645 (0.93)	68 405 (99.07)		0.719
Amazonas	2 505 (0.81)	306 891 (99.19)		0.709
Amapá	543 (0.85)	63 058 (99.15)		0.747
Pará	4 756 (0.86)	548 027 (99.14)		0.675
Rondônia	1 042 (0.97)	106 712 (99.03)		0.715
Roraima	319 (0.76)	41 755 (99.24)		0.732
Tocantins	916 (0.95)	95 567 (99.05)		0.732
**Southeast region**	**56 167 (1.23)**	**4 512 604 (98.77)**	0.0037	
Espírito Santo	2 517 (1.18)	210 847 (98.82)		0.771
Minas Gerais	12 683 (1.23)	1017 576 (98.77)		0.769
Rio de Janeiro	10 278 (1.15)	879 631 (98.85)		0.778
São Paulo	30 689 (1.26)	2 404 550 (98.74)		0.819
**South region**	**18 450 (1.21)**	**1 505 602 (98.79)**	0.0050	
Paraná	7 220 (1.18)	607 078 (98.82)		0.790
Rio Grande do Sul	7 118 (1.28)	547 985 (98.72)		0.779
Santa Catarina	4 112 (1.16)	350 539 (98.84)		0.813

**Total**	**130 001 (1.13)**	**11 421 706 (98.87)**	0.0116	0.761

^**∗**^Ranking of HDI-2014.

**Table 2:** Sociodemographic and obstetrical characteristics of women in twin and single pregnancies, Brazil, SINASC 2011-2014.

**Characteristics**	**Twin pregnancy n (%)**	**Single pregnancy n (%)**	**Cramer V**
**Schooling ** ^**a**^			0.0194
None	6 (0.0)	1 346 (0.01)	
Primary I	1 230 (0.96)	90 740 (0.81)	
Primary II	5 729 (4.48)	483 057 (4.31)	
High school	26 017 (20.36)	2 545 424 (22.72)	
Superior incomplete	65 492 (51.25)	6 256 239 (55.85)	
Superior complete	29 313 (22.94)	1 824 983 (16.29)	
**Age (years) ** ^**b**^			0.0280
< 20	14 225 (10.94)	2 208 896 (19.34)	
20-34	92 963 (71.51)	7 911 889 (69.27)	
35 or above	22 813 (17.55)	1 300 704 (11.39)	
**Marital condition ** ^**c**^			0.0000
With partner	78 626 (61.10)	6 259 141 (55.52)	
Without partner	50 050 (38.90)	5 015 487 (44.48)	
**Skin color/ethnicity ** ^**d**^			0.0000
White	55 354 (44.44)	4 322 827 (39.41)	
Non-white	69 196 (55.56)	6 644 858 (60.59)	
**Parity ** ^**e**^			0.0073
0	42 023 (38.44)	3 931 147 (41.80)	
≥ 1	67 302 (61.56)	5 474 223 (58.20)	
**Number of previous C-sections ** ^**f**^			0.0065
0	75 966 (71.93)	6 768 826 (74.59)	
≥ 1	29 644 (28.07)	2 305 741 (25.41)	
**Number of prenatal visits ** ^**g**^			0.0000
< 7	47 873 (37.22)	4 158 908 (36.77)	
≥ 7	80 752 (62.78)	7 151 902 (63.23)	
**Place of delivery ** ^**h**^			0.0061
Hospital	128 707 (99.01)	11 226 686 (98.30)	
Another health facility	554 (0.43)	89 796 (0.79)	
Home	568 (0.44)	90 158 (0.79)	
Others	164 (0.13)	14 336 (0.13)	

Missing data: (a) 222 131. (b) 217. (c) 148 403. (d) 459 472. (e) 2 037 012. (f) 2 371 530. (g) 112 272. (h) 738.

**Table 3:** Perinatal outcomes from twin pregnancies, Brazil, SINASC 2011-2014.

**Characteristics**	**Twin pregnancy n (%)**		**Single pregnancy n (%)**	**PR (95%CI)**		
**Gestational age at birth ** ^**a**^						
< 32 weeks	12 835 (10.19)		147 877 (1.35)	12.13 (11.93 – 12.33)		
32-36 weeks	54 644 (43.38)		1 011 082 (9.21)	5.17 (5.14 – 5.20)		
≥ 37 weeks	58 478 (46.43)		9 820 746 (89.44)	Ref.		
**Onset of labor ** ^**b**^						
Spontaneous	34 788 (40.39)		1 321 135 (29.97)	Ref.		
Induced	9 396 (11.02)		1 425 306 (32.34)	0.25 (0.24 – 0.26)		
No labor	41 103 (48.19)		1 661 230 (37.69)	0.94 (0.93 – 0.95)		
**Type of birth ** ^**c**^						
Vaginal	24 774 (19.09)		5 092 746 (44.64)	Ref.		
Cesarean section	105 014 (80.91)		6 314 703 (55.36)	3.38 (3.33 – 3.42)		

				**PR (95%CI)**		
	**1st twin n (%)**	**2nd twin n (%)**	**N (%)**	**1st vs single**	**2nd vs single**	**2nd vs 1st**

**Birthweight ** ^**d**^						
< 2500g	75 730 (58.28)	63 213 (61.19)	831 068 (7.28)	17.8 (17.6-18.0)	20.1 (19.8-20.3)	1.1 (1.1-1.1)
≥ 2500g	54 213 (41.72)	40 088 (38.81)	10 582 757 (92.72)	Ref.	Ref.	Ref.
**Presentation ** ^**e**^						
Cephalic	84 095 (77.04)	61 834 (72.14)	6 692 704 (96.32)	Ref.	Ref.	Ref.
Non-cephalic	25 067 (22.96)	23 883 (27.86)	255 596 (3.68)	7.8 (7.7-7.9)	10.1 (9.9-10.3)	1.3 (1.3-1.3)
**Congenital malformation ** ^**f**^						
Yes	1 470 (1.16)	1 144 (1.14)	87 586 (0.79)	1.5 (1.4-1.6)	1.4 (1.3-1.5)	1.0 (0.9-1.1)
Not	124 846 (98.84)	99 406 (98.86)	11 002 510 (99.21)	Ref.	Ref.	Ref.
**5** ^**th**^ ** min Apgar ** ^**g**^						
< 7	3 808 (3.00)	2 787 (2.76)	115 749 (1.05)	2.9 (2.8-3.0)	2.7 (2.6-2.8)	1.1 (1.0-1.1)
≥ 7	122 942 (97.00)	98 283 (97.24)	10 959 760 (98.95)	Ref.	Ref.	Ref.

Missing data: (a) 446 045. (b) 7 058 749. (c) 14 470. (d)9 565. (e) 4 513 455. (f) 3 396 672. (g) 353 305.

**Table 4:** Perinatal outcomes from twin pregnancies according to the presentation of the first twin, Brazil, SINASC 2011-2014.

**Perinatal outcomes**	**Cephalic n (%)**	**Non-cephalic n (%)**	**Cramer V**
**Gestational age at birth ** ^**a**^			0.0384
< 28 weeks	2 662 (3.27)	995 (4.07)	
28-31 weeks	5 204 (6.39)	1 947 (7.97)	
32-36 weeks	35 383 (43.47)	10 864 (44.48)	
≥ 37 weeks	38 141 (46.86)	10 616 (43.47)	
**Onset of labor ** ^**b**^			0.0874
Spontaneous	24 470 (39.27)	9 088 (45.21)	
Induced	7 816 (12.54)	1 310 (6.52)	
No labor	30 021 (48.18)	9 704 (48.27)	
**Type of birth ** ^**c**^			0.1233
Elective cesarean	26 359 (43.20)	8 475 (47.47)	
Intrapartum cesarean	21 265 (34.85)	7 488 (41.94)	
Vaginal 1st/cesarean 2nd	981 (1.61)	226 (1.27)	
Vaginal 1st/vaginal 2nd	12 413 (20.34)	1 663 (9.32)	
**5 min Apgar 1st twin ** ^**d**^			0.0000
< 7	2 282 (2.78)	896 (3.64)	
≥ 7	79 726 (97.22)	23 714 (96.36)	
**5 min Apgar 2nd twin ** ^**e**^			0.0000
< 7	1 454 (2.44)	771 (3.35)	
≥ 7	58 081 (97.56)	22 268 (96.65)	
**5 min Apgar 3rd twin ** ^**f**^			0.0480
< 7	37 (4.99)	12 (2.96)	
≥ 7	704 (95.01)	393 (97.04)	
**5 min Apgar 4th twin ** ^**g**^			0.1016
< 7	3 (13.04)	1 (6.67)	
≥ 7	20 (86.96)	14 (93.33)	

Missing data: (a) 24 189. (b) 47 592. (c) 51 131. (d) 23 383. (e) 18 907. (f) 399. (g) 13.

**Table 5:** Apgar scores at 5 minutes from neonates of twin pregnancies according to the mode of delivery, Brazil, SINASC 2011-2014.

**5 min Apgar score**	**Elective cesarean n (%)**	**Intrapartum cesarean n (%)**	**Vaginal birth for both n (%)**	**Vaginal birth 1st/ Cesarean 2nd n (%)**	**Cramer V**
**1st twin ** ^**a**^					0.1244
< 7	494 (1.38)	539 (1.83)	1 015 (6.57)	52 (3.86)	
≥ 7	35 357 (98.62)	28 892 (98.17)	14 428 (93.43)	1 296 (96.14)	
**2nd twin ** ^**b**^					0.1560
< 7	485 (1.35)	601 (2.04)	1 163 (7.54)	173 (12.86)	
≥ 7	35 353 (98.65)	28 826 (97.96)	14 270 (92.46)	1 172 (87.14)	

Missing data: (a) 47 928. (b) 47 958.

**Table 6:** Factors independentlyassociated with a 5 min Apgar score below 7 among neonates from twin pregnancies: stepwise multiple analyses by nonconditional logistic regression, Brazil, SINASC 2011-2014.

**Variables** [n=58 850]	**Apgar score**	OR_adj_	**95% CI**	**p-value**
Number of prenatal visits <7	Both <7	**2.378**	**1.898-2.978**	**<0.0001**
	1st <7	**1.767**	**1.489-2.097**	**<0.0001**
	2nd <7	**1.517**	**1.294-1.780**	**<0.0001**

Gestational age <32 weeks	Both <7	**30.772**	**21.901-43.236**	**<0.0001**
	1st <7	**10.657**	**8.438-13.460**	**<0.0001**
	2nd <7	**8.029**	**6.465-9.972**	**<0.0001**

Gestational age 32-36 weeks	Both <7	**2.453**	**1.709-3.521**	**<0.0001**
	1st <7	**2.134**	**1.717-2.653**	**<0.0001**
	2nd <7	**2.006**	**1.656-2.431**	**<0.0001**

Intrapartum cesarean	Both <7	**1.266**	**1.012-1.584**	**0.0393**
	1st <7	0.954	0.803-1.133	0.5918
	2nd <7	**1.249**	**1.060-1.471**	**0.0079**

Cesarean for the 2nd twin	Both <7	1.977	0.472-8.287	0.3510
	1st <7	1.115	0.272-4.565	0.8798
	2nd <7	**16.270**	**10.409-25.432**	**<0.0001**

Vaginal birth for both	Both <7	**3.440**	**2.488-4.754**	**<0.0001**
	1st <7	**1.631**	**1.190-2.235**	**0.0023**
	2nd <7	**2.518**	**1.915-3.312**	**<0.0001**

Predictors entering the models: maternal age, schooling, marital status, parity, number of previous cesarean sections, number of prenatal visits, place of birth, gestational age at birth, onset of labor, mode of delivery, ethnicity, and HDI.

## Data Availability

The database from SINASC is available from Internet and information is of public domain.
